# Different complication patterns between anterior and lateral approaches in total ankle arthroplasty: A systematic review and meta‐analysis

**DOI:** 10.1002/jeo2.70774

**Published:** 2026-05-26

**Authors:** Giammarco Gardini, Massimiliano Mosca, Tosca Cerasoli, Giulio Maria Marcheggiani Muccioli, Carlo Capodagli, Marianna Viotto, Cesar de Cesar Netto, Silvio Caravelli

**Affiliations:** ^1^ Istituto Ortopedico Rizzoli U.O. Ortopedia Bentivoglio Bentivoglio Bologna Italy; ^2^ Department of Biomedical and Neuromotor Sciences DIBINEM Alma Mater Studiorum University of Bologna Bologna Italy; ^3^ 2nd Orthopaedic and Traumatologic Clinic IRCCS Rizzoli Orthopaedic Institute Bologna Italy; ^4^ Department of Orthopaedic Surgery, Foot and Ankle Division Duke University School of Medicine Durham North Carolina USA

**Keywords:** ankle prosthesis, anterior approach, revision rates, TAA, total ankle replacement

## Abstract

**Purpose:**

The optimal surgical approach for total ankle arthroplasty (TAA) remains debated. The anterior and lateral transfibular approaches differ in surgical exposure and may influence complication patterns. This systematic review and meta‐analysis aimed to compare revision rates, overall complication rates and specific complication profiles between anterior and lateral approaches in TAA.

**Methods:**

A PRISMA‐compliant systematic review of PubMed, Embase and Scopus databases was performed. Studies reporting complications following primary TAA performed through either approach were included if they met predefined methodological quality criteria (QualSyst score ≥ 75%). Complication rates were compared using Fisher's exact test, and correlations between complication rates and patient or surgical factors were explored.

**Results:**

Fifty‐one studies were included, comprising 7959 TAA procedures (7225 anterior and 734 lateral). Revision rates were higher in the anterior approach group (7.5% vs. 1.9%, *p* < 0.001), as were rates of malleolar fractures (1.7% vs. 0.3%, *p* = 0.001) and aseptic loosening (2.8% vs. 0.14%, *p* < 0.001). Deep infections were reported more frequently in lateral approach cohorts (3.1% vs. 0.57%, *p* < 0.001), although this finding was influenced by outlier studies. Wound complications and polyethylene‐related complications were comparable between approaches. No association was observed between complication rates and patient age, body mass index or surgical time.

**Conclusion:**

Anterior and lateral approaches in TAA appear to be associated with different complication patterns rather than clear differences in overall safety. Both approaches remain valid surgical options, but awareness of their distinct complication profiles may help guide intraoperative precautions and post‐operative surveillance. However, heterogeneity in implant design, follow‐up duration and study methodology limits definitive conclusions regarding the superiority of one approach.

**Level of Evidence:**

Level I, systematic review/meta‐analysis.

**Abbreviations**
BMI: body mass index
TAA: total ankle arthroplasty

## INTRODUCTION

Total ankle arthroplasty (TAA) has become an established and effective treatment for end‐stage ankle arthritis, providing pain relief, functional improvement and preservation of ankle motion [10, 11, 21, 22]. Advances in implant design, instrumentation and surgical techniques have contributed to improved survivorship and expanded indications over the past two decades, with projections indicating a substantial increase in TAA utilization in the coming decades [28].

Despite these developments, several technical aspects of TAA remain debated, including the optimal surgical approach to the ankle joint. The anterior approach represents the most commonly used access in TAA and provides direct exposure of the tibiotalar joint, facilitating implant positioning and component implantation. However, this approach requires dissection through the anterior soft tissues and has been associated with wound‐healing complications due to the relatively limited vascularity of the anterior ankle region and a risk of medial malleolar fractures [17]. The lateral transfibular approach represents an alternative surgical access to the ankle joint. This technique involves fibular osteotomy and lateral exposure of the tibiotalar articulation, allowing direct visualization of the joint surfaces and component positioning. However, this approach introduces different technical challenges and complication patterns, including issues related to fibular osteotomy healing and soft‐tissue management [10, 30, 33].

Given these differences, understanding the complication profiles associated with each surgical approach is clinically relevant for surgical planning and patient counselling. Despite the increasing number of studies comparing these approaches, the literature remains fragmented and heterogeneous, with studies limited by small sample sizes and retrospective designs.

The primary aim of this systematic review and meta‐analysis was to compare revision rates and overall complication rates between anterior and lateral surgical approaches in TAA, while the secondary aim was to evaluate differences in specific complication patterns. The hypothesis was that the two surgical approaches may be associated with different complication patterns. However, given the heterogeneity of the available literature, including differences in implant design, follow‐up duration and study methodology, interpretation of these differences requires careful consideration.

## MATERIALS AND METHODS

**Figure 1:**
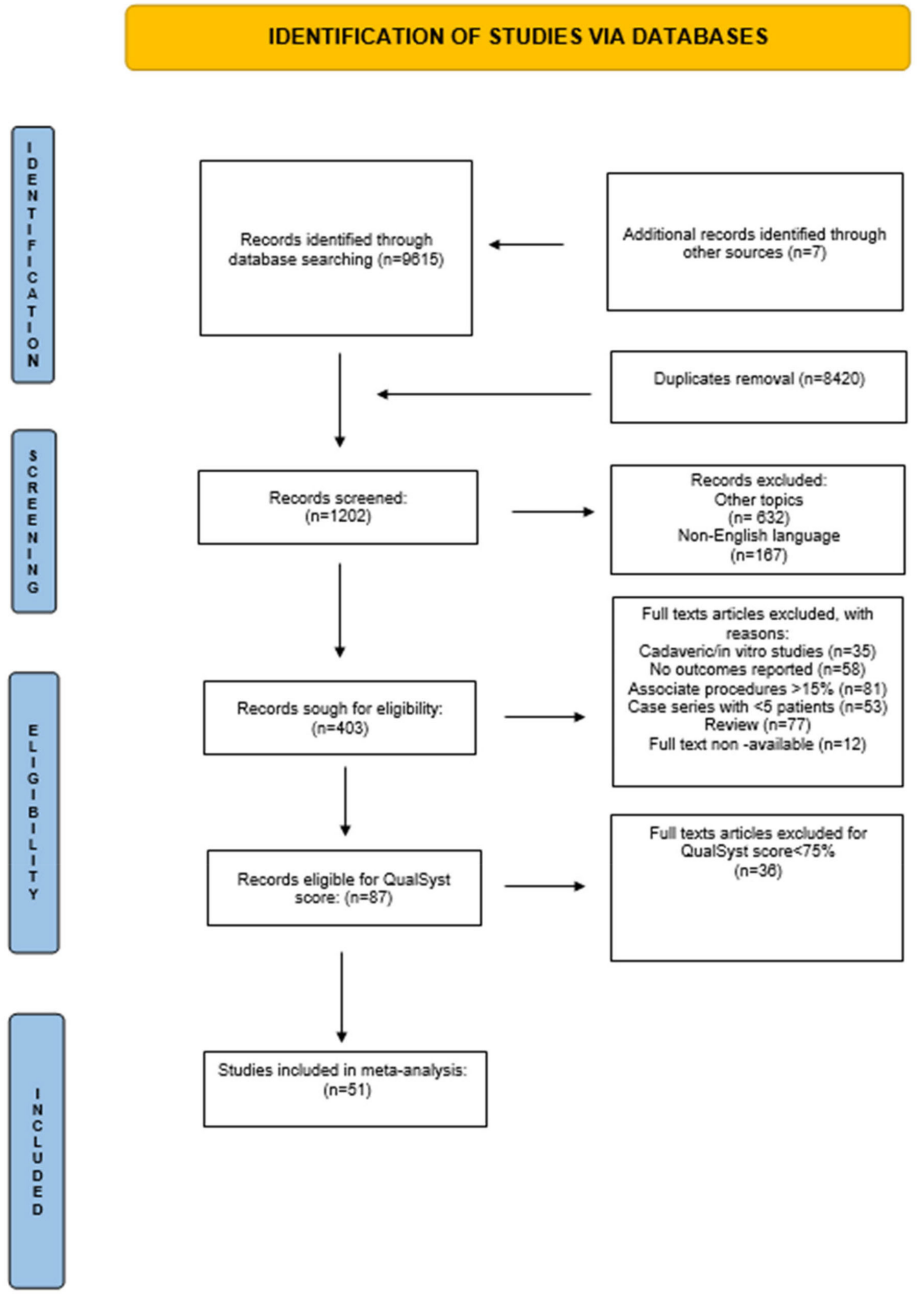
Flow‐chart of the study selection PRISMA process. PRISMA, Preferred Reporting Items for Systematic Reviews and Meta‐Analyses.

A systematic literature search was conducted in PubMed, Embase and Scopus between 1 January 2004 and 1 June 2025, in accordance with the Preferred Reporting Items for Systematic Reviews and Meta‐Analyses (PRISMA) guidelines [22] (Figure 1). The study protocol was registered in the PROSPERO database (ID: CRD420251073944). The aim of this search was to identify studies reporting complications associated with TAA performed through either an anterior or a lateral surgical approach.

The complete search strategy is reported in Supporting Information S1.

Studies were included if they met the following criteria: published in English, with full text available; reported outcomes of primary TAA using a clearly defined surgical approach (anterior or lateral); included at least five patients; provided extractable numerical data regarding revision rates and complications; and involved cohorts in which adjunctive procedures (e.g., osteotomies or ligament reconstructions) accounted for less than 15% of cases (excluding Achilles tendon lengthening or gastrocnemius recession). Studies were excluded if they reported revision TAA procedures, did not specify the surgical approach, or failed to provide extractable outcome data. Case reports, technical notes, conference abstracts and non‐English publications were also excluded. When multiple publications reporting on the same cohort were identified, only the study with the longest available follow‐up was included.

Data extraction was performed independently by two reviewers. For each study, information regarding the number of ankles treated, patient demographics, surgical approach, duration of follow‐up, revision rates and reported complications was collected. Complications of interest included wound complications (defined as delayed wound healing or superficial dehiscence); deep infections; aseptic loosening; periprosthetic cyst formation; and systemic complications (defined as thromboembolic events, particularly deep vein thrombosis and pulmonary embolism). Revision surgery was defined as any surgical procedure involving the removal or replacement of one or more prosthetic components (isolated exchange of polyethylene liner was excluded) or arthrodesis or amputation.

The methodological quality of the included studies was assessed using the QualSyst (KMET) scoring system, which evaluates study design, methodological rigour and reporting quality. Studies with a KMET scores ≥ 75% were considered to have acceptable methodological quality, and were included [14] (the results are depicted in Table S1).

### Statistical analysis

Complication rates between the anterior and lateral approaches were compared using Fisher's exact test for categorical variables. Odds ratios (ORs) and risk ratios (RRs) with 95% confidence intervals (CIs) were calculated to quantify the relative risk of complications between surgical approaches. Spearman's rank correlation coefficient was used to assess potential associations between patient factors, including age, body mass index (BMI) and surgical time, and the overall complication rate. A sensitivity analysis was performed to explore the impact of potential outlier studies. Statistical analyses were performed using Jamovi statistical software, and a *p* value less than 0.05 was considered statistically significant.

## RESULTS

The literature search identified 9622 records, of which 51 studies met the inclusion criteria and were included in the final analysis. Among these, 10 studies were comparative, although two reported outcomes stratified by patient sex and were therefore not treated as comparative analyses in the pooled calculations. Of the included studies, 29 were retrospective, 21 were prospective, and 1 was a randomized controlled trial. The exact distribution of studies per year is reported in Figure S1.

Overall, 7225 TAA performed through an anterior approach, and 734 performed through a lateral transfibular approach were included in the analysis. All analyses were conducted considering the number of implants rather than the number of patients.

Baseline demographic characteristics, follow‐up duration and statistical comparisons are summarized in Table 1.

**Table 1:** Baseline demographic characteristics and follow‐up duration of the included cohorts.

Variable	Overall cohort	Anterior approach	Lateral approach	*p*
Number of implants	7959	7225	734	–
Mean age (years)	62.3 ± 4.2	62.5 ± 3.7	60.0 ± 7.5	>0.05
Mean BMI (kg/m^2^)	28.3 ± 1.6	28.3 ± 1.6	27.0 ± 2.0	>0.05
Mean follow‐up (months)	–	65.7 ± 49.2	33.0 ± 16.1	<0.001

*Note*: Values are reported as mean ± standard deviation.

Abbreviation: BMI, body mass index.

Some complications were reported across the included studies but could not be incorporated in the meta‐analysis due to heterogeneous reporting or insufficient data [2, 3, 4, 5, 6, 7, 9, 18, 23, 27, 29, 31]. These complications are reported in Table S2.

## META‐ANALYSIS

### Revision rate

Revision surgery was defined as any reoperation requiring conversion to ankle arthrodesis, below‐knee amputation, or complete exchange of prosthetic components. Isolated polyethylene insert exchanges were analyzed separately and were not considered revision procedures.

All included studies reported revision rates. A total of 545 revisions were reported among 7225 ankle replacements performed through an anterior approach (7.54%), compared with 14 revisions among 734 replacements performed through a lateral approach (1.91%).

**Figure 2:**
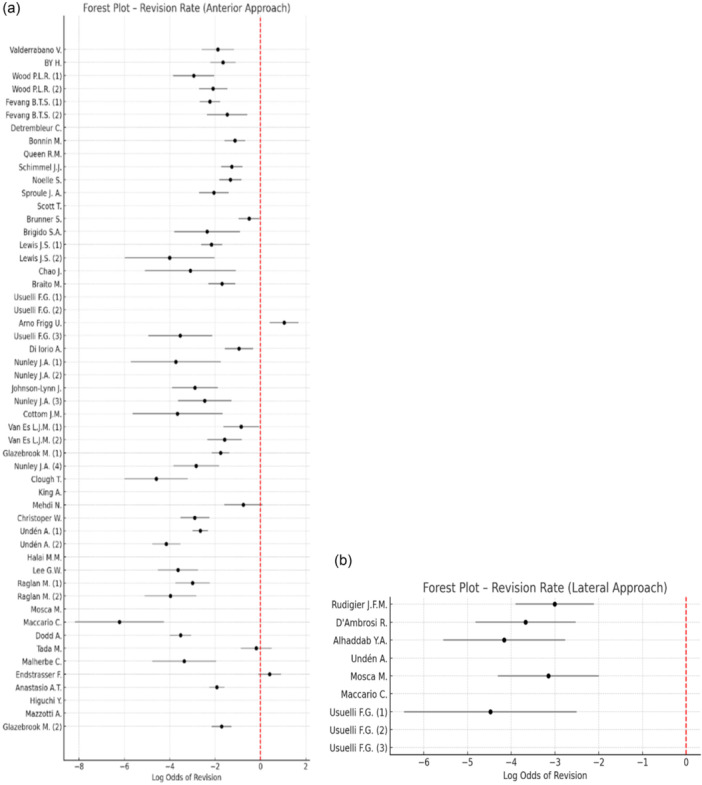
Forest plot showing the log odds of revision for each study. Panel a: Anterior approach in TAA. Panel b: Lateral approach in TAA. Point estimates represent log odds of revision, with 95% confidence intervals shown as horizontal lines. Studies with no reported revisions are plotted with adjusted values to avoid division by zero. The red dashed line indicates the null effect (OR = 1). OR, odds ratio; TAA, total ankle arthroplasty.

A descriptive funnel plot of revision risk according to surgical approach is presented in Figure S2, while the comparison between approaches is illustrated in a descriptive forest plot (Figure 2).

The revision rate was significantly higher in the anterior approach group (*p* < 0.001; Fisher's exact test).

Correlation analysis showed a positive association between follow‐up duration and revision rate in anterior approach studies (Spearman's *ρ* = 0.45, *p* = 0.001). In lateral approach studies, a stronger association was observed (Spearman's *ρ* = 0.80, *p* = 0.009).

### Overall complication rate

Complications occurred in 7711 implants. All included studies reported the total number of complications observed in their cohorts. However, not all studies provided a complete breakdown of individual complication types. Therefore, analyses of specific complications were performed only in studies in which the corresponding outcome was explicitly reported.

**Figure 3:**
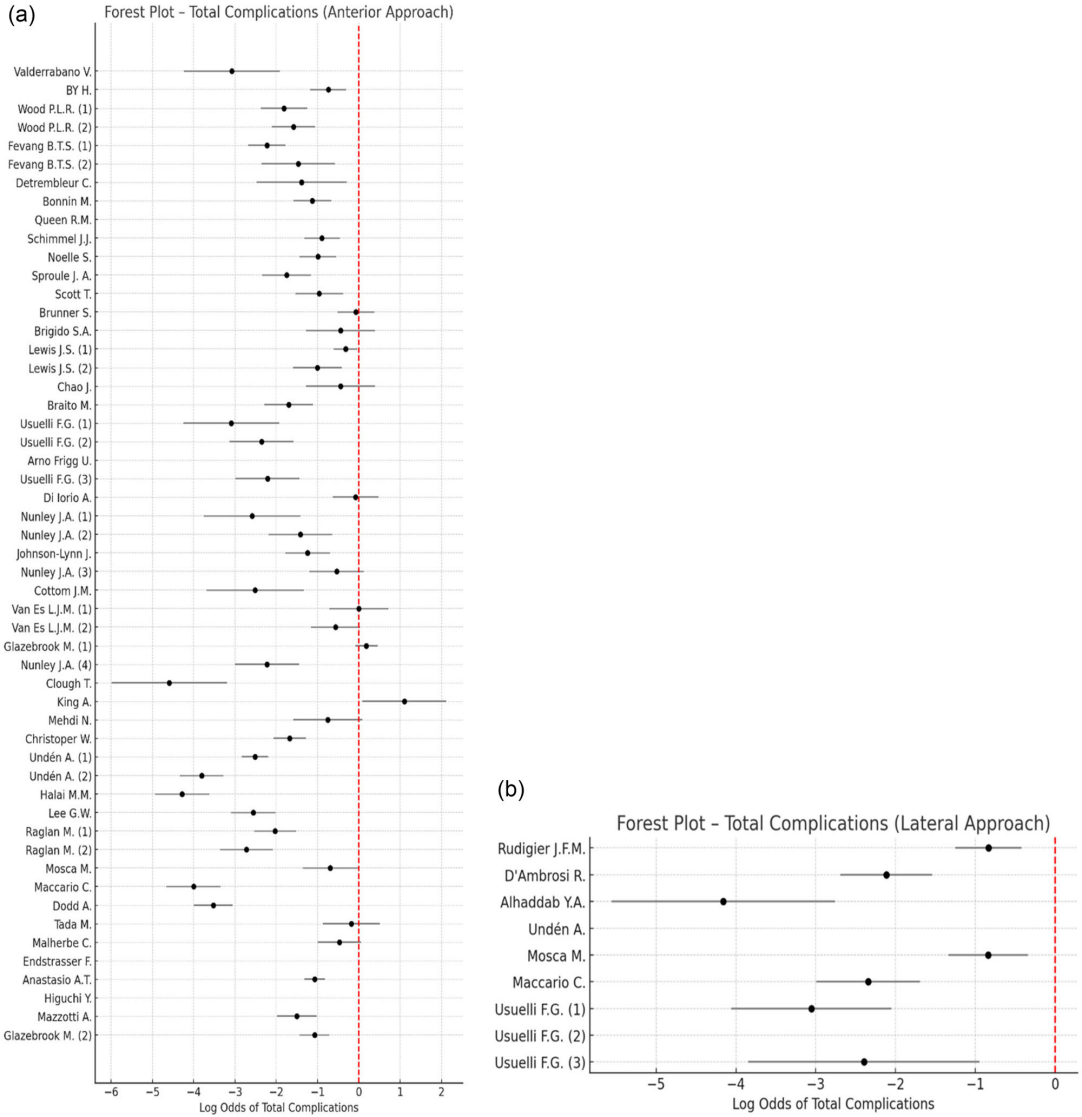
Forest plot showing the log odds of total complications reported in studies. Panel a: Anterior approach in TAA. Panel b: Lateral approach in TAA. Point estimates represent log odds of revision, with 95% confidence intervals shown as horizontal lines. Studies with no reported revisions are plotted with adjusted values to avoid division by zero. The red dashed line indicates the null effect (OR = 1). OR, odds ratio; TAA, total ankle arthroplasty.

A total of 1129 complications were reported among 7225 anterior approach procedures (15.63%) compared with 85 complications among 734 lateral approach procedures (11.58%), as illustrated in a descriptive forest plot (Figure 3) and in the descriptive funnel plot in Figure S3.

The overall complication rate was significantly higher in the anterior approach group (*p* = 0.003).

Correlation analysis between follow‐up duration and reported complications showed a weak but statistically significant association in anterior approach studies (Spearman's *ρ* = 0.31, *p* = 0.027) and a stronger correlation in lateral approach studies (Spearman's *ρ* = 0.76, *p* = 0.017).

### Specific complications

Specific complication types were not uniformly reported across all included studies. Therefore, analyses of individual complications were performed only in studies in which the corresponding outcome was explicitly reported. When a complication is described as ‘reported’ in a study, this indicates that the study specifically evaluated and reported that complication, even if its incidence was zero.

Malleolar fractures were reported in 24 studies, occurring in 125 of 7225 anterior approach procedures (1.7%) and in 2 out of 734 lateral approach procedures (0.3%). The difference between the two approaches was statistically significant (*p* = 0.001).

Wound complications were reported in 30 studies, with a rate of 1.83% in patients treated through an anterior approach and 2.32% in those treated through a lateral approach. This difference was not statistically significant (*p* = 0.319; OR = 0.78; RR = 0.79).

Deep infections were documented in 36 studies, occurring in 47 patients in the anterior approach group (0.57%) and in 23 patients in the lateral approach group (3.13%). The difference between groups was statistically significant (*p* < 0.001). A descriptive funnel plot is reported in Figure S4.

Correlation analysis did not show a significant association between follow‐up duration and the occurrence of malleolar fractures (anterior: Spearman's *ρ* = 0.15, *p* = 0.50; lateral: Spearman's *ρ* = −0.50, *p* = 0.67), wound complications (anterior: Spearman's *ρ* = 0.12, *p* = 0.59; lateral: Spearman's *ρ* = 0.0, *p* = 1.0) or deep infections (anterior: Spearman's *ρ* = 0.23, *p* = 0.24; lateral: Spearman's *ρ* = 0.52, *p* = 0.23).

Mean surgical time was reported in nine studies and was 158.7 min for the anterior approach and 164.8 min for the lateral approach (*p* = 0.883). No significant correlation was found between surgical time and deep infection rates in either approach group.

### Demographics factors

The association between patient demographic variables (reported in all the included studies) and complication rates was evaluated using the mean values reported in the included studies.

No significant correlation was found between mean patient age and overall complication rate in either group (anterior: Pearson *r* = 0.146, *p* = 0.327; lateral: Pearson *r* = 0.581, *p* = 0.121).

Similarly, no significant association was observed between BMI and complication rate in either approach group (anterior: *p* = 0.97; lateral: *p* = 1.00).

### Implant‐related complications

Aseptic loosening was reported in 25 studies, occurring in 205 anterior approach procedures (2.8%) and in 1 lateral approach procedure (0.14%). The difference between the two approaches was statistically significant (*p* < 0.001).

Polyethylene‐related complications were reported in 25 studies. These included rupture or dislocation, and occurred in 171 anterior procedures (2.37%) and in 19 lateral procedures (2.59%), with no statistically significant difference between approaches (*p* = 0.70).

When considering polyethylene insert exchange alone, the incidence was 0.97% in the anterior approach group and 0.41% in the lateral approach group (*p* = 0.155; OR = 2.38).

No significant correlation was found between follow‐up duration and aseptic loosening in either anterior (Spearman's *ρ* = 0.34, *p* = 0.12) or lateral approach studies (Spearman's *ρ* = 0.0, *p* = 1.0).

In anterior approach studies, correlation analysis between follow‐up duration and polyethylene‐related complications showed a positive trend (Spearman's *ρ* = 0.51), although this did not reach statistical significance (*p* = 0.16). For the lateral approach, this analysis could not be performed due to the limited reporting of polyethylene‐related events.

A summary of the results is provided in Table 2.

**Table 2:** Summary of complication rates for anterior versus lateral transfibular approaches.

Complications	Anterior	Lateral	*p*
Revisions	545 (7.54%)	14 (1.91%)	<0.001
Overall complications	1129 (15.63%)	85 (11.58%)	0.003
Malleolar fractures	125 (1.73%)	2 (0.27%)	0.001
Wound complications	132 (1.83%)	17 (2.32%)	0.319
Deep infections	47 (0.65%)	23 (3.13%)	<0.001
Aseptic loosenings	205 (2.84%)	1 (0.14%)	<0.001
Ruptures or dislocations of polyethylene	171 (2.37%)	19 (2.59%)	0.700
Insert exchange only	70 (0.97%)	3 (0.41%)	0.155

*Note*: *p* values were calculated using Fisher's exact test (with continuity correction where needed). A *p* value < 0.05 was considered statistically significant.

## DISCUSSION

This systematic review with meta‐analysis offers one of the most extensive comparisons to date of complication profiles between anterior and lateral approaches in TAA, drawing on data from over 7900 implants across 51 studies. Although previous literature has explored approach‐related outcomes, available evidence remains heterogeneous and often limited by retrospective study designs and variable reporting standards. The present analysis identified differences in complication patterns between the two approaches, including higher rates of revision, malleolar fractures and aseptic loosening in anterior approach series, and a higher reported rate of deep infection in lateral approach cohorts. The higher revision rate observed in the anterior approach group (7.5%) compared with the lateral approach group (1.9%) differs from several previous reports [10, 17, 22].

Several factors may contribute to this discrepancy, including variations in implant design, follow‐up duration, surgeon experience and study design. Anterior approach series span a longer time period and include earlier implant generations, such as the STAR prosthesis, which have historically been associated with higher revision rates. In contrast, many lateral approach studies are more recent and often originate from specialized centres with extensive experience in the transfibular technique [1, 32]. Additionally, although revision rates did not correlate with follow‐up duration, overall complications demonstrated a weak correlation in anterior studies and a stronger correlation in lateral series. Because lateral cohorts generally have shorter follow‐up periods, later complications may be underrepresented, further limiting direct comparisons between the two approaches.

The overall complication rate was also higher in the anterior group (15.6% vs. 11.6%). However, definitions of complications varied considerably across studies, reflecting the broader debate on what constitutes a complication in TAA [25]. Failure is more consistently defined as requiring implant removal, revision, conversion to arthrodesis, or amputation [16]. In the present meta‐analysis, complications intrinsically related to the TAA procedure were analyzed [8], although heterogeneity remains a relevant limitation.

Among specific complications, medial malleolar fractures were significantly more frequent in the anterior cohort, consistent with previous reports [10, 12, 17, 24, 26]. This may be related to medial malleolar manipulation required during anterior exposure, whereas the transfibular approach avoids direct contact with the medial malleolus [15]. Importantly, medial malleolar fractures are often intraoperative, immediately identifiable and typically manageable with screw fixation, rarely affecting long‐term outcomes.

The anterior approach also demonstrated higher rates of aseptic loosening (2.8% vs. 0.14%). This finding is in line with prior reports linking older implant designs and alignment issues with higher loosening rates in anterior approaches [20, 22, 30]. Conversely, the low incidence reported in most lateral approach series may be influenced by several factors, including differences in implant generation, follow‐up duration and the concentration of these procedures in specialized centres with experience in the transfibular technique [15].

Deep infection was reported more frequently in the lateral cohort (3.1% vs. 0.57%). However, this difference was largely influenced by a single study reporting a notably high infection rate (15.1%) [19]. As meta‐analytic estimates may be sensitive to extreme values, these findings should be interpreted cautiously. Indeed, other comparative studies have not consistently demonstrated significant differences in infection risk between surgical approaches [30].

Other complications, such as wound‐healing delays, were comparable between approaches, despite concerns regarding anterior soft‐tissue complications in TAA with an anterior approach [17]. These findings suggest that with contemporary surgical techniques and appropriate patient selection, wound complication rates can be effectively managed in both approaches [10]. Also polyethylene‐related complications (rupture, dislocation or exchange) were similar between groups, supporting previous evidence that these events may depend primarily on implant design rather than on surgical approach.

Correlation analyses did not demonstrate associations between complication rates and patient‐related factors such as age or BMI, nor with surgical time. Although these findings should be considered exploratory, as they are based on aggregated study‐level data rather than individual patient‐level data, they may suggest that advanced age or higher BMI alone should not necessarily preclude patients from being considered candidates for TAA [13].

When interpreting these findings, it is important to consider the intrinsic complexity of TAA. Unlike hip arthroplasty, more than half of patients with end‐stage ankle osteoarthritis present with deformity, malalignment, instability or compromised soft tissues. These factors often require additional corrective procedures during TAA and may influence outcomes independently of the chosen surgical approach. Moreover, the choice of surgical approach in clinical practice is closely linked to implant design. Currently, only one contemporary implant system routinely utilizes a lateral transfibular approach, whereas most commercially available TAA systems are designed for anterior implantation. As a result, comparisons between surgical approaches may partly reflect differences in implant generation, biomechanics and surgical instrumentation rather than the approach itself. This interdependence represents one of the main limitations of the available literature and complicates attempts to isolate the independent effect of the surgical approach on complication rates [15, 31].

This review is further limited by the predominance of retrospective studies, variability in complication definitions, and the substantial sample‐size imbalance between approaches (>7200 anterior vs. <750 lateral). This imbalance may influence comparative estimates and should be considered when interpreting differences between the two surgical approaches. Although appropriate statistical methods were applied, rare‐event outcomes and potential outlier studies must be interpreted cautiously. Another methodological aspect concerns the quality threshold applied during study selection. Only studies with a QualSyst (KMET) score ≥75% were included in the analysis, a threshold commonly considered indicative of acceptable methodological quality according to the original description of the tool by Kmet and Lee [14]. This criterion was applied to reduce the influence of studies with substantial methodological limitations on the pooled estimates. Another limitation relates to the heterogeneity in complication reporting across studies. While all included studies reported the total number of complications, several publications did not provide a detailed breakdown of specific complication types. As a consequence, analyses of individual complications were restricted to studies explicitly reporting those outcomes, which may have reduced the available sample size for certain analyses.

## CONCLUSION

This systematic review suggests that anterior and lateral approaches in TAA are associated with different complication patterns rather than clear differences in overall safety. Anterior approach series showed higher rates of revision, malleolar fractures and aseptic loosening, whereas lateral approach cohorts demonstrated a higher reported rate of deep infection, although this finding was influenced by outlier studies.

From a clinical perspective, these findings suggest that both approaches remain valid surgical options, but they may require different perioperative awareness and follow‐up strategies. Surgeons should be aware of the distinct complication profiles associated with each technique and tailor intraoperative precautions and post‐operative surveillance accordingly.

However, these observations must be interpreted in the context of important confounding factors, including implant design, follow‐up duration and heterogeneity in study methodology. Consequently, the current literature does not allow definitive conclusions regarding the superiority of one surgical approach over the other.

Future prospective studies with standardized complication reporting and implant‐specific analyses will be necessary to better clarify the independent role of surgical approach in TAA outcomes.

## AUTHOR CONTRIBUTIONS

*Conceptualization*: Giammarco Gardini, Massimiliano Mosca, Silvio Caravelli and Tosca Cerasoli. *Methodology*: Giammarco Gardini, Tosca Cerasoli and Marianna Viotto. *Literature search and data curation*: Giammarco Gardini and Tosca Cerasoli. *Formal analysis*: Tosca Cerasoli. *Investigation*: Giammarco Gardini, Tosca Cerasoli, Carlo Capodagli and Marianna Viotto. *Writing—original draft*: Giammarco Gardini, Tosca Cerasoli. *Writing—review and editing*: Giulio Maria Marcheggiani Muccioli, Silvio Caravelli and Cesar de Cesar Netto. *Supervision*: Giulio Maria Marcheggiani Muccioli, Massimiliano Mosca, Silvio Caravelli and Cesar de Cesar Netto. All authors have read and approved the final manuscript.

## FUNDING INFORMATION

The authors have no funding to report.

## CONFLICT OF INTEREST STATEMENT

The authors declare no conflicts of interest.

## ETHICS STATEMENT

The authors have nothing to report.

## Supporting information

Figure S1. Distribution of studies per year.
Figure S2. Funnel plot of revision risk for total ankle arthroplasty by surgical approach. Each point represents an individual study. The x‐axis displays the log odds of revision, and the y‐axis shows the standard error. Blue circles represent studies using the lateral approach, while orange triangles represent the anterior approach. A greater number and wider dispersion of anterior studies.
Figure S3. Funnel plot of overall complication risk for total ankle arthroplasty according to surgical approach. Each point represents a single study. The x‐axis shows the log odds of complications, and the y‐axis displays the standard error. The blue circle corresponds to the lateral approach, while the orange triangle corresponds to the anterior approach. The lateral approach studies appear more numerous and widely dispersed, suggesting a broader variability in complication rates. In contrast, anterior approach studies are fewer and more symmetrically distributed.
Figure S4. Funnel plot of deep infection per study. Each cross represents a single study. The x‐axis shows the log odds of deep infections rate, and the y‐axis displays the standard error. The orange cross corresponds to the lateral approach, while the yellow cross corresponds to the anterior approach.
Table S1. Methodological quality assessment of the included studies using the QualSyst (KMET) scoring system and complete bibliography. Studies with a score ≥75% were considered to have acceptable methodological quality and were included in the analysis.
Table S2. Additional complications reported in the included studies that were not included in the meta‐analysis due to heterogeneous or incomplete reporting across studies.

## Data Availability

All the data are previously published since it is a meta‐analysis.
