# Supplementary material for: Different complication patterns between anterior and lateral approaches in total ankle arthroplasty: A systematic review and meta‐analysis
Source: J Exp Orthop. 2026 May 26;13(2):e70774. doi: 10.1002/jeo2.70774 (PMC13239087; doi:10.1002/jeo2.70774)
Supplement: Supplementary file 1 — Figure S1. Distribution of studies per year. Figure S2. Funnel plot of revision risk for total ankle arthroplasty by surgical approach. Each point represents an individual study. The x‐axis displays the log odds of revision, and the y‐axis shows the standard error. Blue circles represent studies using the lateral approach, while orange triangles represent the anterior approach. A greater number and wider dispersion of anterior studies. Figure S3. Funnel plot of overall complication risk for total ankle arthroplasty according to surgical approach. Each point represents a single study. The x‐axis shows the log odds of complications, and the y‐axis displays the standard error. The blue circle corresponds to the lateral approach, while the orange triangle corresponds to the anterior approach. The lateral approach studies appear more numerous and widely dispersed, suggesting a broader variability in complication rates. In contrast, anterior approach studies are fewer and more symmetrically distributed. Figure S4. Funnel plot of deep infection per study. Each cross represents a single study. The x‐axis shows the log odds of deep infections rate, and the y‐axis displays the standard error. The orange cross corresponds to the lateral approach, while the yellow cross corresponds to the anterior approach. Table S1. Methodological quality assessment of the included studies using the QualSyst (KMET) scoring system and complete bibliography. Studies with a score ≥75% were considered to have acceptable methodological quality and were included in the analysis. Table S2. Additional complications reported in the included studies that were not included in the meta‐analysis due to heterogeneous or incomplete reporting across studies. [file JEO2-13-e70774-s001.docx]

**Supplementary Materials**

**Complete search strategy:**  [(replacement OR arthroplasty OR prosthesis) AND ankle AND ((anterior OR lateral) AND approach) AND (complications OR failures OR adverse)].

Supplementary Table S1. Methodological quality assessment of the included studies using the QualSyst (KMET) scoring system and complete bibliography. Studies with a score ≥75% were considered to have acceptable methodological quality and were included in the analysis.

| **First Author** | **Year** | **Study Type** | **QualSyst Score (%)** | **Quality Level** |
| --- | --- | --- | --- | --- |
| Valderrabano V.[1] | 2004 | Retrospective | 80 | Moderate |
| Rudigier J.F.M.[2] | 2005 | Retrospective | 80 | Moderate |
| BY H.[3] | 2006 | Prospective | 90 | High |
| Wood P.L.R.[4] | 2009 | Randomized | 95 | High |
| Fevang B.T.S.[5] | 2009 | Retrospective | 80 | Moderate |
| Detrembleur C.[6] | 2009 | Prospective | 90 | High |
| Bonnin M.[7] | 2010 | Retrospective | 80 | Moderate |
| Queen R.M.[8] | 2012 | Prospective | 90 | High |
| Schimmel J.J.[9] | 2013 | Retrospective | 80 | Moderate |
| Noelle S.[10] | 2013 | Prospective | 90 | High |
| Sproule J. A.[11] | 2013 | Retrospective | 80 | Moderate |
| Bleazey S. T.[12] | 2013 | Retrospective | 80 | Moderate |
| Brunner S.[13] | 2013 | Prospective | 90 | High |
| Brigido S.A.[14] | 2014 | Prospective | 90 | High |
| Lewis J.S.[15] | 2015 | Prospective | 90 | High |
| Chao J.[16] | 2015 | Prospective | 90 | High |
| Braito M.[17] | 2015 | Retrospective | 80 | Moderate |
| Usuelli F.G.[18] | 2017 | Retrospective | 80 | Moderate |
| Arno Frigg U. [19] | 2017 | Retrospective | 80 | Moderate |
| Usuelli F.G.[20] | 2019 | Retrospective | 80 | Moderate |
| Di Iorio A.[21] | 2017 | Prospective | 90 | High |
| Johnson-Lynn J.[22] | 2017 | Retrospective | 80 | Moderate |
| Nunley J.A.[23] | 2018 | Retrospective | 80 | Moderate |
| Nunley J.A.[24] | 2018 | Retrospective | 80 | Moderate |
| Clough T.[25] | 2019 | Prospective | 90 | High |
| King A.[26] | 2019 | Prospective | 90 | High |
| Mehdi N.[27] | 2019 | Prospective | 90 | High |
| Usuelli F.G.[28] | 2019 | Retrospective | 80 | Moderate |
| Usuelli F.G.[29] | 2019 | Prospective | 90 | High |
| Christoper W.[30] | 2019 | Retrospective | 80 | Moderate |
| Undén A.[31] | 2020 | Retrospective | 80 | Moderate |
| Halai M.M.[32] | 2020 | Prospective | 90 | High |
| Lee G.W.[33] | 2020 | Retrospective | 80 | Moderate |
| Raglan M.[34] | 2020 | Retrospective | 80 | Moderate |
| Mosca M.[35] | 2020 | Prospective | 90 | High |
| Maccario C.[36] | 2020 | Retrospective | 80 | Moderate |
| Townshend D.N.[37] | 2021 | Prospective | 90 | High |
| Dodd A.[38] | 2021 | Prospective | 90 | High |
| D'Ambrosi R.[39] | 2021 | Retrospective | 80 | Moderate |
| Tada M.[40] | 2022 | Prospective | 90 | High |
| Malherbe C.[41] | 2023 | Retrospective | 80 | Moderate |
| Endstrasser F.[42] | 2023 | Retrospective | 80 | Moderate |
| Alhaddab Y.A.[43] | 2023 | Retrospective | 80 | Moderate |
| Cottom J.M.[44] | 2023 | Prospective | 90 | High |
| Van Es L.J.M.[45] | 2024 | Retrospective | 80 | Moderate |
| Anastasio A.T.[46] | 2024 | Retrospective | 80 | Moderate |
| Higuchi Y.[47] | 2024 | Retrospective | 80 | Moderate |
| Mazzotti A.[48] | 2024 | Retrospective | 80 | Moderate |
| Glazebrook M.[49] | 2024 | Retrospective | 80 | Moderate |
| Protheroe D.[50] | 2024 | Prospective | 90 | High |
| Glazebrook M.[51] | 2025 | Retrospective | 80 | Moderate |

Bibliography

1. Valderrabano, V.; Hintermann, B.; Dick, W. Scandinavian Total Ankle Replacement: A 3.7-Year Average Followup of 65 Patients. *Clin. Orthop. Relat. Res.* **2004**, 47–56.

2. Rudigier, J. Ankle Replacement by the Cementless ESKA Endoprosthesis. *Tech. Foot Ankle Surg.* **2005**, *4*, 125–136.

3. Doets, H.C.; Brand, R.; Nelissen, R.G.H.H. Total Ankle Arthroplasty in Inflammatory Joint Disease with Use of Two  Mobile-Bearing Designs. *J. Bone Joint Surg. Am.* **2006**, *88*, 1272–1284, doi:10.2106/JBJS.E.00414.

4. Wood, P.L.R.; Sutton, C.; Mishra, V.; Suneja, R. A Randomised, Controlled Trial of Two Mobile-Bearing Total Ankle Replacements. *J. Bone Joint Surg. Br.* **2009**, *91*, 69–74, doi:10.1302/0301-620X.91B1.21346.

5. Fevang, B.-T.S.; Lie, S.A.; Havelin, L.I.; Brun, J.G.; Skredderstuen, A.; Furnes, O. 257 Ankle Arthroplasties Performed in Norway between 1994 and 2005. *Acta Orthop.* **2007**, *78*, 575–583, doi:10.1080/17453670710014257.

6. Detrembleur, C.; Leemrijse, T. The Effects of Total Ankle Replacement on Gait Disability: Analysis of Energetic  and Mechanical Variables. *Gait Posture* **2009**, *29*, 270–274, doi:10.1016/j.gaitpost.2008.09.009.

7. Bonnin, M.; Gaudot, F.; Laurent, J.-R.; Ellis, S.; Colombier, J.-A.; Judet, T. The Salto Total Ankle Arthroplasty: Survivorship and Analysis of Failures at 7 to  11 Years. *Clin. Orthop. Relat. Res.* **2011**, *469*, 225–236, doi:10.1007/s11999-010-1453-y.

8. Queen, R.M.; Biasio, J.C. De; Butler, R.J.; DeOrio, J.K.; Easley, M.E.; Nunley, J.A. Changes in Pain, Function, and Gait Mechanics Two Years Following Total Ankle Arthroplasty Performed with Two Modern Fixed-Bearing Prostheses. *Foot Ankle Int.* **2012**, *33*, 535–542, doi:10.3113/FAI.2012.0535.

9. Schimmel, J.J.P.; Walschot, L.H.B.; Louwerens, J.W.K. Comparison of the Short-Term Results of the First and Last 50 Scandinavian Total  Ankle Replacements: Assessment of the Learning Curve in a Consecutive Series. *Foot Ankle Int.* **2014**, *35*, 326–333, doi:10.1177/1071100713518187.

10. Noelle, S.; Egidy, C.C.; Cross, M.B.; Gebauer, M.; Klauser, W. Complication Rates after Total Ankle Arthroplasty in One Hundred Consecutive  Prostheses. *Int. Orthop.* **2013**, *37*, 1789–1794, doi:10.1007/s00264-013-1971-9.

11. Sproule, J.A.; Chin, T.; Amin, A.; Daniels, T.; Younger, A.S.; Boyd, G.; Glazebrook, M.A. Clinical and Radiographic Outcomes of the Mobility Total Ankle Arthroplasty  System: Early Results from a Prospective Multicenter Study. *Foot Ankle Int.* **2013**, *34*, 491–497, doi:10.1177/1071100713477610.

12. Bleazey, S.T.; Brigido, S.A.; Protzman, N.M. Perioperative Complications of a Modular Stem Fixed-Bearing Total Ankle  Replacement with Intramedullary Guidance. *J. Foot Ankle Surg.* **2013**, *52*, 36–41, doi:10.1053/j.jfas.2012.10.009.

13. Brunner, S.; Barg, A.; Knupp, M.; Zwicky, L.; Kapron, A.L.; Valderrabano, V.; Hintermann, B. The Scandinavian Total Ankle Replacement: Long-Term, Eleven to Fifteen-Year,  Survivorship Analysis of the Prosthesis in Seventy-Two Consecutive Patients. *J. Bone Joint Surg. Am.* **2013**, *95*, 711–718, doi:10.2106/JBJS.K.01580.

14. Brigido, S.A.; Galli, M.M.; Bleazey, S.T.; Protzman, N.M. Modular Stem Fixed-Bearing Total Ankle Replacement: Prospective Results of 23 Consecutive Cases with 3-Year Follow-Up. *The Journal of Foot and Ankle Surgery* **2014**, *53*, 692–699, doi:10.1053/j.jfas.2014.04.001.

15. Lewis, J.S.J.; Green, C.L.; Adams, S.B.J.; Easley, M.E.; DeOrio, J.K.; Nunley, J.A. Comparison of First- and Second-Generation Fixed-Bearing Total Ankle Arthroplasty  Using a Modular Intramedullary Tibial Component. *Foot Ankle Int.* **2015**, *36*, 881–890, doi:10.1177/1071100715576568.

16. Chao, J.; Choi, J.H.; Grear, B.J.; Tenenbaum, S.; Bariteau, J.T.; Brodsky, J.W. Early Radiographic and Clinical Results of Salto Total Ankle Arthroplasty as a  Fixed-Bearing Device. *Foot Ankle Surg.* **2015**, *21*, 91–96, doi:10.1016/j.fas.2014.09.012.

17. Braito, M.; Dammerer, D.; Reinthaler, A.; Kaufmann, G.; Huber, D.; Biedermann, R. Effect of Coronal and Sagittal Alignment on Outcome After Mobile-Bearing Total  Ankle Replacement. *Foot Ankle Int.* **2015**, *36*, 1029–1037, doi:10.1177/1071100715583383.

18. Usuelli, F.G.; Manzi, L.; Brusaferri, G.; Neher, R.E.; Guelfi, M.; Maccario, C. Sagittal Tibiotalar Translation and Clinical Outcomes in Mobile and Fixed-Bearing  Total Ankle Replacement. *Foot Ankle Surg.* **2017**, *23*, 95–101, doi:10.1016/j.fas.2016.08.005.

19. Frigg, A.; Germann, U.; Huber, M.; Horisberger, M. Survival of the Scandinavian Total Ankle Replacement (STAR): Results of Ten to  Nineteen Years Follow-Up. *Int. Orthop.* **2017**, *41*, 2075–2082, doi:10.1007/s00264-017-3583-2.

20. Usuelli, F.G.; Indino, C.; Maccario, C.; Manzi, L.; Liuni, F.M.; Vulcano, E. Infections in Primary Total Ankle Replacement: Anterior Approach versus Lateral  Transfibular Approach. *Foot Ankle Surg.* **2019**, *25*, 19–23, doi:10.1016/j.fas.2017.07.643.

21. Di Iorio, A.; Viste, A.; Fessy, M.H.; Besse, J.L. The AES Total Ankle Arthroplasty Analysis of Failures and Survivorship at Ten  Years. *Int. Orthop.* **2017**, *41*, 2525–2533, doi:10.1007/s00264-017-3605-0.

22. Johnson-Lynn, S.E.; Ramaskandhan, J.; Siddique, M.S. The Effect of Patient Age and Diagnosis on the 5-Year Outcomes of Mobile-Bearing  Total Ankle Replacement. *Foot (Edinb).* **2018**, *36*, 1–5, doi:10.1016/j.foot.2018.07.001.

23. J.A. Nunley, J. DeOrio, M. Easley, M. Stewart, S.A. Mid-Term (5–10 Year) Results of the Salto Talaris Total Ankle Arthroplasty. *Foot & ankle surgery*, doi:10.1016/j.fas.2017.07.245.

24. Nunley, J.A.; Adams, S.B.; Easley, M.E.; DeOrio, J.K. Prospective Randomized Trial Comparing Mobile-Bearing and Fixed-Bearing Total  Ankle Replacement. *Foot Ankle Int.* **2019**, *40*, 1239–1248, doi:10.1177/1071100719879680.

25. Clough, T.; Bodo, K.; Majeed, H.; Davenport, J.; Karski, M. Survivorship and Long-Term Outcome of a Consecutive Series of 200 Scandinavian  Total Ankle Replacement (STAR) Implants. *Bone Joint J.* **2019**, *101-B*, 47–54, doi:10.1302/0301-620X.101B1.BJJ-2018-0801.R1.

26. King, A.; Bali, N.; Kassam, A.-A.; Hughes, A.; Talbot, N.; Sharpe, I. Early Outcomes and Radiographic Alignment of the Infinity Total Ankle Replacement  with a Minimum of Two Year Follow-up Data. *Foot Ankle Surg.* **2019**, *25*, 826–833, doi:10.1016/j.fas.2018.11.007.

27. Mehdi, N.; Bernasconi, A.; Laborde, J.; Lintz, F. Comparison of 25 Ankle Arthrodeses and 25 Replacements at 67 Months’ Follow-Up. *Orthop. Traumatol. Surg. Res.* **2019**, *105*, 139–144, doi:10.1016/j.otsr.2018.10.014.

28. Usuelli, F.G.; Maccario, C.; Granata, F.; Indino, C.; Vakhshori, V.; Tan, E.W. Clinical and Radiological Outcomes of Transfibular Total Ankle Arthroplasty. *Foot Ankle Int.* **2019**, *40*, 24–33, doi:10.1177/1071100718798851.

29. Usuelli FG, Indino C, Manzi L, M.C.; D’Ambrosi R, G.C. Sport and Physical Activities in Total Ankle Replacement: Mobile- and Fix-Bearing. *Arch Trauma Res* **2017**, *6*, 6.

30. Reb, C.W.; Watson, B.C.; Fidler, C.M.; Van Dyke, B.; Hyer, C.F.; Berlet, G.C.; Prissel, M.A. Anterior Ankle Incision Wound Complications Between Total Ankle Replacement and  Ankle Arthrodesis: A Matched Cohort Study. *J. Foot Ankle Surg.* **2021**, *60*, 47–50, doi:10.1053/j.jfas.2020.04.015.

31. Undén, A.; Jehpsson, L.; Kamrad, I.; Carlsson, Å.; Henricson, A.; Karlsson, M.K.; Rosengren, B.E. Better Implant Survival with Modern Ankle Prosthetic Designs: 1,226 Total Ankle  Prostheses Followed for up to 20 Years in the Swedish Ankle Registry. *Acta Orthop.* **2020**, *91*, 191–196, doi:10.1080/17453674.2019.1709312.

32. Halai, M.M.; Pinsker, E.; Daniels, T.R. Effect of Novel Anteromedial Approach on Wound Complications Following Ankle  Arthroplasty. *Foot Ankle Int.* **2020**, *41*, 1198–1205, doi:10.1177/1071100720937247.

33. Lee, G.-W.; Lee, K.-B. Does Gender Influence the Outcomes of Total Ankle Arthroplasty in Patients with  Ankle Osteoarthritis? *J. Orthop. Surg. Res.* **2020**, *15*, 207, doi:10.1186/s13018-020-01731-5.

34. Raglan, M.; Machin, J.T.; Cro, S.; Taylor, A.; Dhar, S. Total Ankle Replacement : Comparison of the Outcomes of STAR and Mobility. *Acta Orthop. Belg.* **2020**, *86*, 109–114.

35. Mosca, M.; Caravelli, S.; Vocale, E.; Maitan, N.; Grassi, A.; Massimi, S.; Fuiano, M.; Zaffagnini, S. Clinical-Radiological Outcomes and Complications after Total Ankle Replacement  through a Lateral Transfibular Approach: A Retrospective Evaluation at a Mid-Term Follow-Up. *Int. Orthop.* **2021**, *45*, 437–443, doi:10.1007/s00264-020-04709-4.

36. Maccario, C.; Tan, E.W.; Di Silvestri, C.A.; Indino, C.; Kang, H.P.; Usuelli, F.G. Learning Curve Assessment for Total Ankle Replacement Using the Transfibular Approach. *Foot and Ankle Surgery* **2021**, *27*, 129–137, doi:https://doi.org/10.1016/j.fas.2020.03.005.

37. Townshend, D.N.; Bing, A.J.F.; Clough, T.M.; Sharpe, I.T.; Goldberg, A. Early Experience and Patient-Reported Outcomes of 503 INFINITY Total Ankle  Arthroplasties. *Bone Joint J.* **2021**, *103-B*, 1270–1276, doi:10.1302/0301-620X.103B7.BJJ-2020-2058.R2.

38. Dodd, A.; Pinsker, E.; Younger, A.S.E.; Penner, M.J.; Wing, K.J.; Dryden, P.J.; Glazebrook, M.; Daniels, T.R. Sex Differences in End-Stage Ankle Arthritis and Following Total Ankle  Replacement or Ankle Arthrodesis. *J. Bone Joint Surg. Am.* **2022**, *104*, 221–228, doi:10.2106/JBJS.21.00287.

39. D’Ambrosi, R.; Tiusanen, H.T.; Ellington, J.K.; Kraus, F.; Younger, A.; Usuelli, F.G. Fixed-Bearing Trabecular Metal Total Ankle Arthroplasty Using the Transfibular  Approach for End-Stage Ankle Osteoarthritis: An International Non-Designer Multicenter Prospective Cohort Study. *JB JS Open Access* **2022**, *7*, doi:10.2106/JBJS.OA.21.00143.

40. Tada, M.; Inui, K.; Okano, T.; Koike, T. Comparison of Clinical and Radiographic Results of Mobile-Bearing Total Ankle  Arthroplasty between Osteoarthritis and Rheumatoid Arthritis. *Mod. Rheumatol.* **2023**, *33*, 1104–1109, doi:10.1093/mr/roac123.

41. Malherbe, C.; Deleu, P.-A.; Devos Bevernage, B.; Birch, I.; Maldague, P.; Gombault, V.; Putzeys, P.; Leemrijse, T. Early-Term Results of the Cadence Total Ankle Prosthesis: An European Noninventor  Study. *Foot Ankle Int.* **2023**, *44*, 1–12, doi:10.1177/10711007221136539.

42. Endstrasser, F.; Lindtner, R.A.; Landegger, A.; Wagner, M.; Eichinger, M.; Schönthaler, H.; Kaufmann, G.; Brunner, A. Midterm Results of AAA Ankle Arthroplasty. *Foot Ankle Int.* **2023**, *44*, 983–991, doi:10.1177/10711007231186375.

43. Alhaddab, Y.A.; Mittal, R.; Symes, M.J.; Wines, A.P. Rate of Infection and Causative Organisms in a Lateral Approach Total Ankle  Replacement. *Foot Ankle Spec.* **2025**, *18*, 251–257, doi:10.1177/19386400231184960.

44. Cottom, J.M.; Badell, J.S.; Wolf, J. Two-Year Outcomes After Total Ankle Replacement With a Novel Fixed-Bearing  Implant By a Single Surgeon Non-Inventor. *J. Foot Ankle Surg.* **2024**, *63*, 337–344, doi:10.1053/j.jfas.2024.01.001.

45. van Es, L.J.M.; Sierevelt, I.N.; Kerkhoffs, G.M.M.J.; Haverkamp, D. Analyzing Learning Curve Effects: Total Ankle Replacement Design Switch and  Long-Term Survival. *J. Foot Ankle Surg.* **2024**, *63*, 593–597, doi:10.1053/j.jfas.2024.06.002.

46. Anastasio, A.T.; Adams, S.B.; DeOrio, J.K.; Easley, M.E.; Nunley, J.A.; Lee, D.-O. Comparison of Radiographic Talar Loosening Rates Between Salto-Talaris and INBONE  II. *Foot Ankle Int.* **2024**, *45*, 60–66, doi:10.1177/10711007231209763.

47. Higuchi, Y.; Hirao, M.; Noguchi, T.; Etani, Y.; Ebina, K.; Okamura, G.; Tsuboi, H.; Miyama, A.; Takahi, K.; Takami, K.; et al. Early Mobilization of Dorsiflexion from 3 Days after Cemented Total Ankle  Arthroplasty with Modified Antero-Lateral Approach. *J. Orthop. Sci.* **2024**, *29*, 874–879, doi:10.1016/j.jos.2023.04.010.

48. Mazzotti, A.; Artioli, E.; Giannini, I.; Zielli, S.O.; Arceri, A.; Langone, L.; Faldini, C. Drain versus No Drain after Total Ankle Arthroplasty: Are There Any Differences  in Complications Rate? *Arch. Orthop. Trauma Surg.* **2024**, *144*, 1071–1076, doi:10.1007/s00402-023-05165-6.

49. Glazebrook, M.; Balasubramaniam, U.; Walls, A.; Younger, A.S.E.; Penner, M.; Wing, K.; Dryden, P.J.; Daniels, T.R. Outcomes of Total Ankle Replacement Versus Ankle Arthrodesis for the Treatment of  End-Stage Ankle Arthritis: A Concise Follow-up, at a Minimum of 10 Years, of a Previous Report. *J. Bone Joint Surg. Am.* **2025**, *107*, 552–557, doi:10.2106/JBJS.24.00361.

50. Protheroe, D.; Gadgil, A.; Davies, G. Hintegra® Total Ankle Replacement. Five and 10 Year Survivorship Analysis,  Clinical Outcomes, Complications and Satisfaction Rates. *J. Clin. Orthop. Trauma* **2025**, *64*, 102950, doi:10.1016/j.jcot.2025.102950.

51. Glazebrook M, Balasubramaniam U, Walls A, Younger A S E, Penner M, Wing K, Dryden P J, D.T.R. Outcomes of Total Ankle Replacement Versus Ankle Arthrodesis for the Treatment of End-Stage Ankle Arthritis. **2024**.

Supplementary Table S2. Additional complications reported in the included studies that were not included in the meta-analysis due to heterogeneous or incomplete reporting across studies.

| Complication | Total Number Of Events | Number Of Studies Reporting The Complication | Studies |
| --- | --- | --- | --- |
| Osteolysis Without Revision Surgery | 22 | 4 | Chao 2015; Protheroe 2025; Sproule 2013; Wood 2009 |
| Edge Loading | 14 | 2 | Wood 2009; Fevang 2007 |
| Osteolytic Cysts | 40 | 3 | Bonnin 2011; Brunner 2013; Di Iorio 2017 |
| Unexplained Pain | 14 | 5 | Sproule 2013; Fevang 2007; Bonnin 2011; Brunner 2013; Noelle 2013 |
| Impingement | 9 | 2 | Bleazey 2013; Frigg 2017 |
| Systemic Complications (Dvt/Pe) | 4 | 2 | Bleazey 2013; Brigido 2014 |

Figure S1. Distribution of studies per year


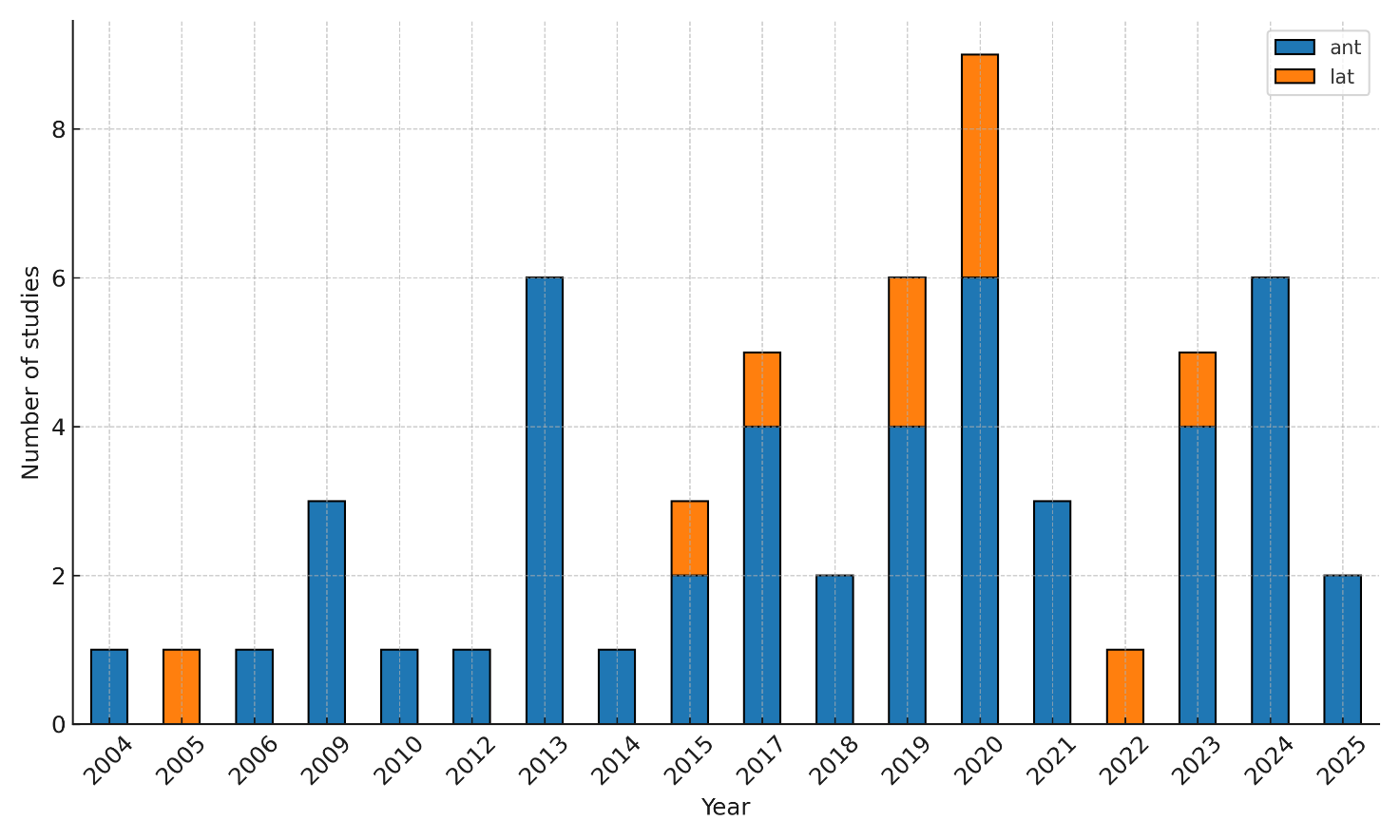


Figure S2. Funnel plot of revision risk for total ankle arthroplasty by surgical approach.
Each point represents an individual study. The x-axis displays the log odds of revision, and the y-axis shows the standard error. Blue circles represent studies using the lateral approach, while orange triangles represent the anterior approach. A greater number and wider dispersion of anterior studies.


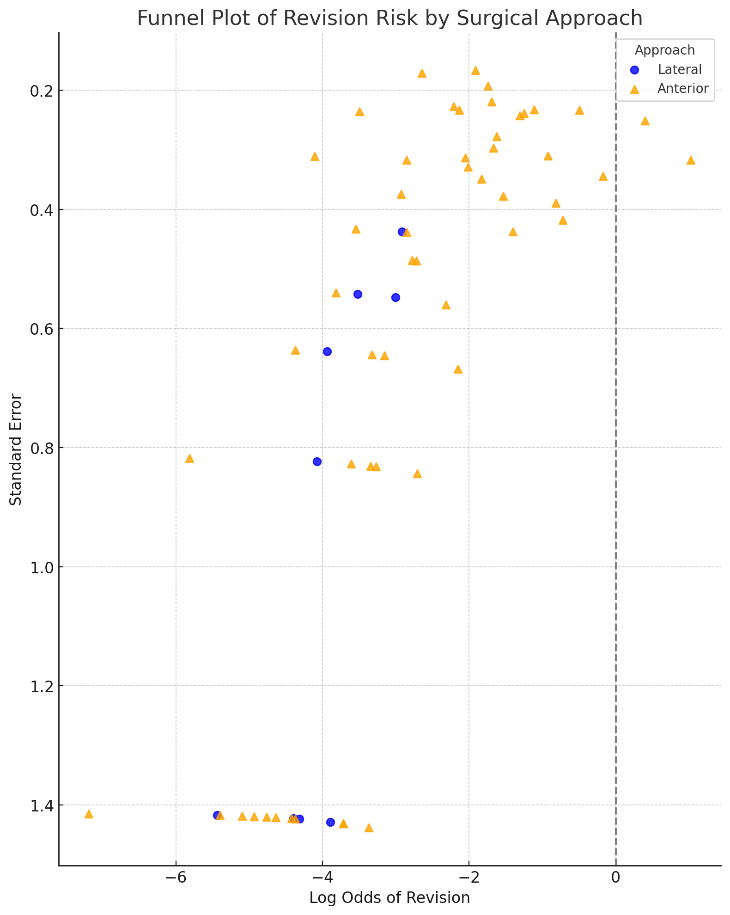


Figure S3. Funnel plot of overall complication risk for total ankle arthroplasty according to surgical approach.
Each point represents a single study. The x-axis shows the log odds of complications, and the y-axis displays the standard error. The blue circle corresponds to the lateral approach, while the orange triangle corresponds to the anterior approach. The lateral approach studies appear more numerous and widely dispersed, suggesting a broader variability in complication rates. In contrast, anterior approach studies are fewer and more symmetrically distributed.


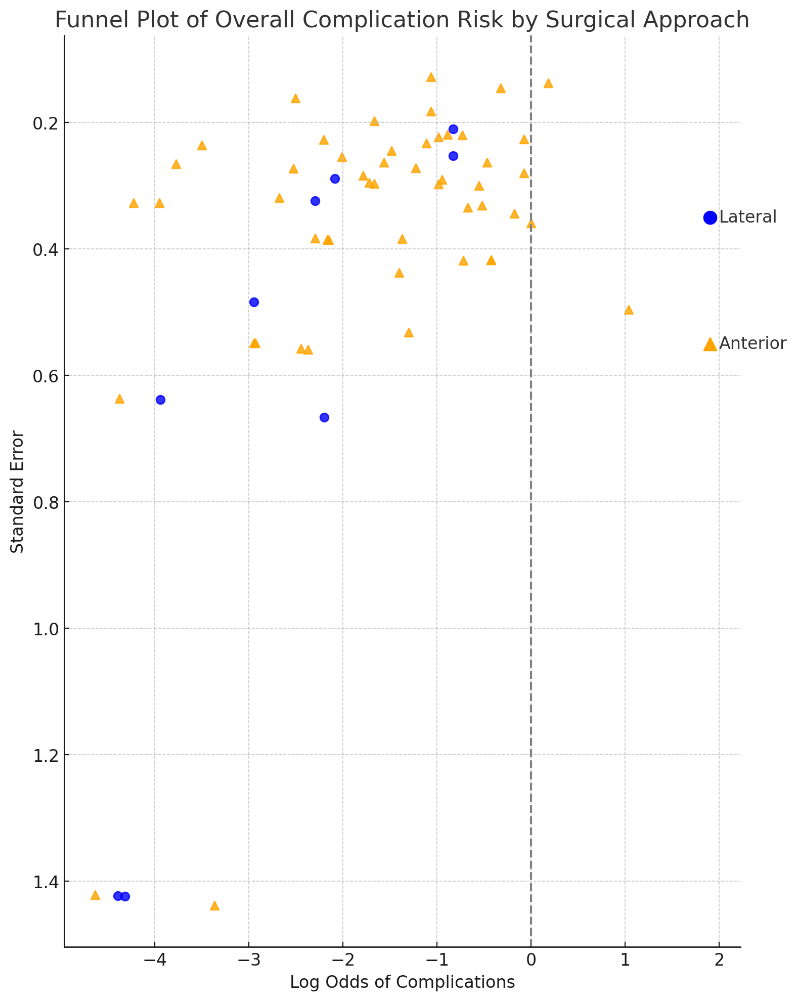


Figure S4. Funnel plot of deep infection per study. Each cross represents a single study. The x-axis shows the log odds of deep infections rate, and the y-axis displays the standard error. The orange cross corresponds to the lateral approach, while the yellow cross corresponds to the anterior approach.

**
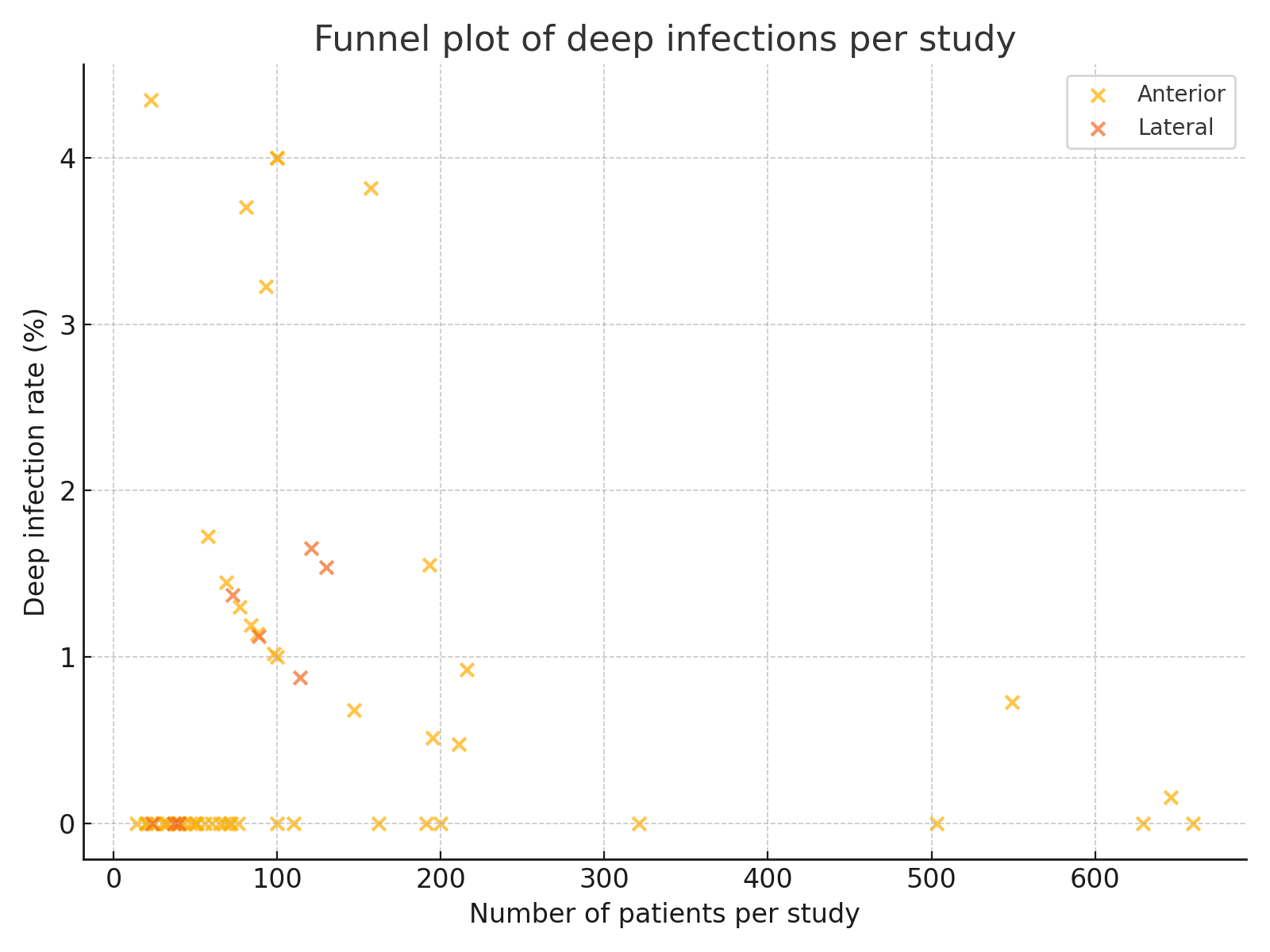
**
